# Trends in lawyer use in road traffic injury compensation claims

**DOI:** 10.1371/journal.pone.0231025

**Published:** 2020-04-06

**Authors:** Clare E. Scollay, Janneke Berecki-Gisolf, Genevieve M. Grant

**Affiliations:** 1 Faculty of Law, Monash University, Melbourne, Victoria, Australia; 2 Monash University Accident Research Centre, Monash University, Melbourne, Victoria, Australia; Tongii University, CHINA

## Abstract

Injury compensation claimants use legal services to help them navigate compensation schemes, including accessing benefits and resolving disputes. Little is known, however, about the extent of lawyer use by compensation claimants, including changes over time. This paper presents findings from one of the largest empirical investigations of lawyer use in an injury compensation setting to date. Using evidence from more than 275,000 claims in the road traffic injury scheme in the state of Victoria, Australia, this study examines the prevalence of, and changes in, lawyer use between 2000 and 2015. The analysis identifies a significant increase in the use of lawyers in the scheme, and explores possible explanations. This study provides critical insights into lawyer use in compensation settings: the steep increase in lawyer involvement has both access to justice and financial implications for compensation schemes, given the associations between lawyer use, claimant outcomes, and long-term scheme viability.

## Introduction

### Compensation schemes as a response to road traffic injuries

Road traffic crashes are a leading cause of death worldwide, resulting in over 1.25 million fatalities and 50 million injuries per annum [1]. Individuals injured in road traffic crashes have a range of physical, psychological, and legal needs that often persist over long periods of time [2, 3]. These needs also often result in long-term employment and financial difficulties [4–6] with profound personal, social, and economic consequences [7].

In many middle-income countries, legislatures have responded to the needs of those injured in road traffic crashes through the establishment of statutory injury compensation schemes and modifications to traditional tort litigation [8]. In Australia, the main function of compensation schemes is to distribute benefits to injured persons in order to return them to work and health as efficiently and effectively as possible [9–11]. Schemes are typically charged with fulfilling this function whilst also minimising costs to society and delivering public health benefits through safety promotion and crash prevention activities [9, 10].

There is considerable variation in the nature and extent of the benefits provided by road traffic injury compensation schemes. In Australia, for example, all state and territory jurisdictions now provide no-fault benefits in the form of long-term care and support to persons who sustain significant and permanent injuries [12]. For other injured persons, there is variable access to a range of benefits, which are provided on a no-fault or fault basis [12]. No-fault benefits are generally accessible by all injured persons irrespective of their contribution to the crash circumstances, whilst access to fault-based benefits is restricted to injured persons who can establish that their injuries were caused by the negligence of another party and exceed a minimum severity [8, 13].

### Lawyer use in road traffic injury compensation schemes

Lawyers play an instrumental role in enabling claimants to access their legal entitlements, particularly when they might struggle to do so otherwise. Injured claimants may engage lawyers to navigate the complex and unfamiliar compensation process, access benefits, and resolve disputes [14–16]. The role of lawyers in facilitating claimant access to entitlements is particularly important in injury compensation schemes, given insurers’ fundamental conflict of interest between fairly compensating claimants, and maximising profit or safeguarding the ongoing financial viability of the scheme. Insurers also have several advantages over claimants in that they control the resources that claimants are seeking to access [15], and so hold the balance of power in the relationship [17]. In addition, insurers are experts in compensation and tort processes as they are ‘repeat players’ [18]. Specialist lawyers may also be repeat players: their engagement can equalise some of the power and knowledge imbalances that exist between claimants and insurers, and prevent claimants from being unduly disadvantaged in compensation settings.

Despite the crucial role played by lawyers in compensation schemes, there has been little quantitative investigation into use of their services after road traffic injury [9, 19]. The few studies that have examined lawyer use have used it as a predictor rather than an outcome [9, 19]. These studies have found a relationship between lawyer use and negative recovery outcomes, including physical and mental ill-health [20–22]; longer treatment times [20]; delays in, and lack of, claim closure [23–26]; and lower perceived fairness of the compensation process [16]. However, these studies are observational and cannot establish causality and, as a result, questions have been raised about the direction of this relationship [27, 28], the mechanisms linking lawyer use to claimant outcomes [19], and the influence of external factors such as the tendency of lawyers to select cases based on economic viability [9].

### Study aims

In contrast, there has been minimal examination of lawyer use as an outcome in compensation schemes. As such, the prevalence and drivers of lawyer use in compensation schemes are not well understood [27]. Equally, whilst lawyer use is a measure of aspects of scheme performance, it is challenging to interpret: routine use of lawyers in a scheme may indicate a complex claims process in which claimants struggle to understand and access their rights without assistance. At the same time, the absence of lawyers from a scheme may indicate financial and institutional barriers to claimants accessing legal services, and challenges to access to justice as a result. Other interpretations are also possible. There is a pressing need for studies that examine lawyer use as an outcome to begin to build an evidence base around whether, when, and how claimants are using legal services, and how this is changing over time [19, 27, 29]. This study addresses this need by investigating lawyer use in 276,546 claims in the road traffic injury compensation scheme in the state of Victoria, Australia. It examines (a) the number and proportion of claims involving lawyer use in the scheme, (b) how these have changed over time, and (c) whether observed changes are due to variations in contextual factors (such as changes in numbers of road traffic crashes and serious injuries arising from crashes). This will begin to develop our understanding of how claimants are using lawyers in the Victorian scheme, and the implications of this for claimant access to entitlements and justice, and scheme performance.

### Research context: Road traffic injury compensation in Victoria

In Victoria, individuals are eligible to claim compensation from the state insurer (the Transport Accident Commission [TAC]) if they are injured in a land-based transport crash involving a car, motorcycle, bus, train, or tram [30]. Compensation is also available to persons injured interstate if the crash involved a Victorian-registered motor vehicle, and to the dependents of a person killed in a transport crash if the deceased person would have been entitled to compensation.

Injured persons are eligible for income replacement, medical, rehabilitation, and lifetime care costs, irrespective of fault. If a claimant has a permanent physical or psychological whole-person impairment assessed as being above 10 percent, they may also be entitled to an impairment lump sum payment [30]. Impairment is assessed through independent medical examinations that occur 18 months after the crash or once the injury has stabilised, whichever is latest. To access impairment lump sum payments, injured persons need to lodge a claim with the TAC within six years of injury.

If a claimant has injuries that are classified as serious and can establish that another party was negligent in their crash circumstances, they may also be entitled to ‘common law’ (i.e., traditional tort) damages. Injuries are classified as serious if they result in a permanent impairment of 30 percent or more or satisfy a narrative test of their effects on claimants’ abilities to engage in valued activities [30]. To access common law benefits, injured persons need to lodge a claim with the TAC within six years of injury.

The TAC scheme is similar to the blended no-fault and tort scheme that operates in the Canadian province of Ontario, in which injured persons are able to claim compensation for medical, rehabilitation, and care costs on a no-fault basis (up to a maximum cap), as well as tort damages where another party was at fault in their crash and their injuries are both permanent and serious [31]. The TAC scheme is also similar to the no-fault schemes with limited tort options defined by verbal thresholds that operate in some US states (including Florida, Michigan, New Jersey, New York, and Pennsylvania); in these schemes, benefits are provided on a no-fault basis and suits are prevented unless the injuries incurred are defined as serious under state statute [32, 33].

## Materials and methods

### Data sources and selection

At the time of this study, administrative data relating to claims lodged with the TAC were housed in the deidentified Compensation Research Database (CRD) at Monash University [34]. Data for claims relating to crashes that occurred between 1 January 2000 and 31 December 2015 were extracted from the CRD for use in this study (N = 294,817 claims made by N = 282,097 claimants). January 2000 was selected as the lower limit as lawyer use data were incomplete prior to this date. December 2015 was selected as the upper limit as data for the 2016 calendar year were incomplete at time of analysis. Claims for crashes that occurred interstate and for fatalities were removed from the dataset (N = 18,271 claims made by N = 17,610 claimants). Interstate claims were excluded because entitlement to common law damages is determined in accordance with the law of the state or territory in which the crash occurred, rather than Victorian law. Claims for fatalities were excluded because these relate to the needs of surviving dependents, which are manifestly different to the needs of injured persons.

Statewide data on the total number of crashes occurring each calendar year between 2006 and 2015 were obtained from VicRoads; data prior to 2006 were not publically available [35]. Statewide data on the total number of hospitalisations from transport crashes occurring each calendar year between 2000 and 2015 were obtained from the Victorian Injury Surveillance Unit (VISU) [36]. Hospitalisations were included if the patient was a Victorian resident, the principal diagnosis was for an injury, and the cause of this injury was a transport accident (ICD-10-AM code S00-T75 or T79). To capture incident hospitalisations only, transfers from another hospital or within the same hospital were excluded, as were readmissions for treatment within 30 days of initial hospitalisation. Finally, base population information was extracted from publically available datasets produced by the Australian Bureau of Statistics (ABS) [37].

Institutional ethics approval for the study was received from the Monash University Human Research Ethic Committee (project number: 2016–0816).

### Data definitions and analysis strategy

Data were analysed using SAS 9.4 for Windows and SPSS Statistics 23.0.

#### Trends in contextual factors

Several analyses were undertaken to examine trends in contextual factors such as crashes, hospitalisations from crashes, TAC claims involving hospitalisations, and crash type over time. First, incidence rates for crashes and hospitalisations from crashes were calculated by crash year and expressed as annual rate per 100,000 Victorian state population. TAC claims involving hospitalisation within one week of injury were calculated by crash year and expressed as a proportion of all hospitalisations from crashes. The proportion of single- and multi- vehicle crashes were also calculated by crash year. Second, to determine trends in the absolute numbers of crashes, hospitalisations from crashes, and TAC claims involving hospitalisations over time, univariate linear regression analyses were conducted. These analyses examined the association between year and the outcome of interest (e.g., crashes). Third, to determine trends in rates per 100,000 population of crashes and hospitalisations from crashes, as well as trends in TAC claims that involved hospitalisation as a proportion of all hospitalisations, Poisson regression analyses (with the exposed population size as offset) were conducted. The Poisson distribution was assumed given the count nature of the data: the use of Poisson models for count data is common in both compensation [38–43] and crash likelihood prediction [44] studies. In this study, the regression analyses examined the association between year and the outcome of interest.

#### Trends in lawyer use

Several analyses were undertaken to examine trends in lawyer use over time. Claims were summarised by year and outcome according to three groups: (1) no-fault claims without impairment lump sum components (‘no-fault claims’), (2) no-fault claims with impairment lump sum components, and (3) common law claims. If a claim involved both impairment lump sum and common law components, it was classified as a common law claim. Common law claims can take several years to finalise, leading most claimants to access no-fault and impairment lump sum benefits in the interim. No-fault impairment lump sum claims were identified through the presence of an impairment lump sum (or annuity) payment, whilst common law claims were identified through the presence of a common law flag.

For each group, the total number of claims and the proportion of claims involving lawyer use were calculated by crash year. Lawyer use was identified through the presence of either a solicitor engagement date or a payment for legal services. The solicitor engagement date was calculated by the TAC as the earliest of the following dates: (a) the solicitor start date entered into the system by TAC legal staff, (b) dates derived from legal documents, or (c) dates derived from references to solicitors in claim notes.

To standardise the follow-up period across claims, a limited sample with a four-year follow-up period was created for data used in regression analyses. This was done by redefining lawyer use as the presence of either a solicitor engagement date or a payment for legal services within four years of the crash date. In addition, claims with less than four years of follow-up data (i.e., claims relating to crashes that occurred between 1 January 2012 and 31 December 2015) were excluded from the dataset. Four years was selected as the follow-up period based on the distribution of time from crash date to lawyer use; this period captured 85.0 percent of lawyer use for crashes occurring between 1 January 2000 and 31 December 2011.

To test for bias in this limited sample, Chi-Square tests were conducted to examine differences between claims with first lawyer use prior to four years post-crash, and claims with first lawyer use after four years post-crash for claims relating to crashes that occurred between 1 January 2000 and 31 December 2011 (for other examples of the use of Chi-Square tests to compare sample characteristics in compensation studies, see [39, 45]). The variables used in the Chi-square analyses were those used in the multivariable logistic regression analyses (see below).

To determine trends in lawyer use over time, univariate logistic regression analyses were conducted using the limited sample. These analyses examined the association between crash year and lawyer use in each of the three groups. To determine whether trends in lawyer use over time were due to changes in contextual factors, multivariable logistic regressions were conducted using the limited sample. These analyses examined the association between crash year and lawyer use in each of the three groups, controlling for crash type (single- or multi-vehicle), number of hospitalisation days (as a proxy for injury severity), gender, age, SES (IRSAD State Decile) [46] and remoteness (primary ARIA grouping) [47]. Year was modelled as a continuous variable in these analyses (this is a common practice: for some recent examples see [48–52]).

## Results

### Sample characteristics

The final sample (N = 276,546 claims made by N = 264,487 claimants) included more males (51.9%) than females (48.1%), and the greatest proportion of claimants were in the 15 to <25 age category (22.4%). Most claimants lived in a major city (73.3%), and approximately half were within the top five SES deciles (52.2%). Most claimants were injured in a crash that involved more than one vehicle (62.1%), and were not hospitalised due to their injuries (63.5%).

### Trends in contextual factors

The annual frequencies and rates per 100,000 population of all Victorian crashes and hospitalisations from crashes are presented in Table 1. Although the absolute number of crashes increased significantly over the study period (F(1,8) = 10.67, p<0.05), the annual rate per 100,000 population decreased slightly, by -1.2% per annum (95% CI -0.8% to -1.5%, p<0.0001). Similarly, although the absolute number of transport accident related hospital admissions did not change significantly over the study period (F(1,14) = 3.47, p = 0.08), the annual rate per 100,000 population decreased slightly, by -1.0% per annum (95% CI -0.4% to -1.6%, p<0.001). The number of TAC claims that involved hospitalisations also did not change significantly over the study period (F(1,14) = 0.09, p = 0.77), although the percentage of TAC claims that involved hospitalisations as a proportion of all hospitalisations decreased by -0.6% per annum (95% CI -0.1% to -1.2%, p<0.05).

**Table 1:** Trends in crashes, hospitalisations from crashes, and TAC claims involving hospitalisations from 2000 to 2015.

Time (Crash Year)	Crashes* N (Rate per 100,000 Population)	Hospitalisations from Crashes^ N (Rate per 100,000 Population)	TAC Claims Involving Hospitalisations^#^ N	TAC Claims Involving Hospitalisations/ Hospitalisations from Crashes %
**2000**		7,613 (162)	5,951	78.2%
**2001**		8,890 (186)	6,306	70.9%
**2002**		8,674 (180)	6,011	69.3%
**2003**		8,502 (174)	6,044	71.1%
**2004**		8,415 (170)	5,585	66.4%
**2005**		8,974 (180)	6,153	68.6%
**2006**	13,428 (265)	8,752 (172)	5,665	64.7%
**2007**	13,483 (261)	9,202 (178)	6,041	65.6%
**2008**	14,036 (266)	9,299 (176)	5,947	64.0%
**2009**	13,703 (254)	8,953 (166)	5,734	64.0%
**2010**	13,476 (246)	9,040 (165)	5,663	62.6%
**2011**	13,829 (249)	9,648 (174)	6,049	62.7%
**2012**	13,720 (243)	8,488 (150)	5,590	65.9%
**2013**	13,853 (241)	8,147 (142)	5,592	68.6%
**2014**	14,249 (243)	8,844 (151)	6,109	69.1%
**2015**	14,379 (241)	9,626 (161)	6,442	66.9%

* Data obtained from VicRoads

^ Data obtained from VISU

^#^ Data obtained from the CRD

The proportion of TAC claims that arose from single-vehicle compared to multi-vehicle crashes remained stable over the study period (M = 36.5%, SD = 4.3%).

### Trends in lawyer use

#### Trends in lawyer use (overall sample)

Lawyer use between 2000 and 2015 is presented in Table 2. Overall, 39,955 claims (14.4% of the sample) involved lawyer use. As data for 2014 and 2015 were incomplete (due to the length of time required for injuries to stabilise before impairment lump sum and common law claims can be lodged), trends in lawyer use from 2000 to 2013 only are discussed below.

**Table 2:** Trends in lawyer use in no-fault claims, no-fault impairment lump sum claims, common law claims, and all claims from 2000 to 2015.

Time (Crash Year)	No-Fault Claims		No-Fault Impairment Lump Sum Claims		Common Law Claims		All Claims	
	Claims N	Claims with Lawyer Involvement N (%)	Claims N	Claims with Lawyer Involvement N (%)	Claims N	Claims with Lawyer Involvement N (%)	Claims N	Claims with Lawyer Involvement N (%)
**2000**	16,440	389 (2.4%)	452	252 (55.8%)	1,380	1,317 (95.4%)	18,272	1,958 (10.7%)
**2001**	16,033	365 (2.3%)	406	230 (56.7%)	1,367	1,321 (96.6%)	17,806	1,916 (10.8%)
**2002**	15,635	380 (2.4%)	372	223 (59.9%)	1,206	1,172 (97.2%)	17,213	1,775 (10.3%)
**2003**	15,687	479 (3.1%)	430	280 (65.1%)	1,043	1,004 (96.3%)	17,160	1,763 (10.3%)
**2004**	14,678	550 (3.7%)	470	329 (70.0%)	971	941 (96.9%)	16,119	1,820 (11.3%)
**2005**	15,391	610 (4.0%)	565	397 (70.3%)	1,105	1,085 (98.2%)	17,061	2,092 (12.3%)
**2006**	14,532	596 (4.1%)	590	419 (71.0%)	1,214	1,197 (98.6%)	16,336	2,212 (13.5%)
**2007**	14,989	701 (4.7%)	569	431 (75.7%)	1,304	1,293 (99.2%)	16,862	2,425 (14.4%)
**2008**	14,310	812 (5.7%)	579	494 (85.3%)	1,346	1,335 (99.2%)	16,235	2,641 (16.3%)
**2009**	15,043	1,122 (7.5%)	608	531 (87.3%)	1,369	1,359 (99.3%)	17,020	3,012 (17.7%)
**2010**	14,773	1,101 (7.5%)	570	524 (91.9%)	1,320	1,312 (99.4%)	16,663	2,937 (17.6%)
**2011**	15,046	1,728 (11.5%)	620	574 (92.6%)	1,268	1,262 (99.5%)	16,934	3,564 (21.0%)
**2012^**	14,761	2,111 (14.3%)	536	510 (95.1%)	1,055	1,053 (99.8%)	16,352	3,674 (22.5%)
**2013^**	15,898	2,222 (14.0%)	415	397 (95.7%)	836	830 (99.3%)	17,149	3,449 (20.1%)
2014*	18,946	1,927 (10.2%)	272	252 (92.6%)	490	478 (97.6%)	19,708	2,657 (13.5%)
2015*	19,484	1,906 (9.8%)	70	59 (84.3%)	102	95 (93.1%)	19,656	2,060 (10.5%)
**Total**	251,646	16,999	7,524	5,902	17,376	17,054	276,546	39,955

^ The proportion of claims with lawyer use in 2012 and 2013 is likely to be an underestimate, as a four-year follow-up period captures 85 percent of lawyer use

* Data for 2014 and 2015 are incomplete due to the length of time required before impairment level can be assessed and claims can be lodged (either 18 months or until the injury has stabilised, whichever is latest)

The proportion of claims that involved lawyer use increased from 10.7% in 2000 to 20.1% in 2013 (a difference of 9.4% [95% CI 8.7% to 10.2%]). The no-fault claims group had the smallest proportion of claims that involved lawyer use. This proportion increased from 2.4% in 2000 to 14.0% in 2013 (a difference of 11.9% [95% CI 11.0% to 12.2%]). The no-fault impairment lump sum claims group had the next greatest proportion of claims that involved lawyer use. This proportion increased from 55.8% in 2000 to 95.7% in 2013 (a difference of 39.9% [95% CI 34.8% to 44.8%]). The common law claims group had the greatest proportion of claims that involved lawyer use. This proportion increased from 95.4% in 2000 to 99.3% in 2013 (a difference of 3.9% [95% CI 2.6% to 5.1%]).

The median time from crash date to initial lawyer use for all claims relating to accidents that occurred between 1 January 2000 and 31 December 2013 was 455 days for the sample as a whole, 329 days for the no-fault claims group, 507 days for the no-fault impairment lump sum claims group, and 540 days for the common law claims group.

#### Differences between claims with lawyer use before vs. after four years post-crash

To standardise the follow-up period across claims, a limited sample with a four-year follow-up period was created. In this sample, claims relating to crashes that occurred after 31 December 2011 were removed, and the definition of lawyer use was restricted to use within four years post-crash. Of the 203,681 claims made by 198,321 claimants that related to crashes occurring between 1 January 2000 and 31 December 2011, 28,115 claims made by 27,331 claimants involved lawyer use. Restricting the definition of lawyer use to use within four years post-crash reduced this number to 23,899 claims made by 23,413 claimants. It also introduced some bias, as there were significant differences in gender (χ2(1) = 11.48, p<0.01), age (χ2(8) = 335.84, p<0.01), SES (χ2(9) = 23.44, p<0.01), crash type (χ2(1) = 25.76, p<0.01) and injury severity (χ2(3) = 418.75, p<0.01) between claims with lawyer use within four years post-crash and claims with lawyer use after four years post-crash. Specifically, claims with lawyer use within four years post-crash were relatively more likely to be made by claimants who were female (43.6% vs. 40.8%), aged 45 years or above (42.9% vs. 32.9%), within the top five SES deciles (i.e., socio-economically advantaged; 48.2% vs. 46.5%), involved in multi-vehicle crashes (60.2% vs. 56.0%) and hospitalised for two or more days (56.5% vs. 41.4%) compared to claims with lawyer use after four years post-crash.

#### Trends in lawyer use (claims with lawyer use within four years post-crash)

Logistic regression models were used to determine whether lawyer involvement increased over time; the results are presented in Table 3. In all groups, crash year was positively associated with lawyer use: lawyer use per claim increased during the study period. This association remained after adjusting for crash type, length of hospital stay (as a proxy for injury severity), gender, age, SES, and remoteness. In the overall sample, the association remained after also adjusting for claim type. For the full models, see S1 Appendix of Table A1.

**Table 3:** Univariate and multivariate logistic regression results.

Group	Total N (N With Outcome)	Variable	Crude Model Odds Ratio (95% Confidence Interval)	p-value	Adjusted Model Odds Ratio (95% Confidence Interval)*	p-value
**No-Fault Claims**	181,064 (7,642)	Crash Year	1.252 (95% CI 1.242 to 1.262)	p<0.001	1.259 (95% CI 1.249 to 1.269)	p<0.001
**No-Fault Impairment Lump Sum Claims**	5,153 (3,360)	Crash Year	1.310 (95% CI 1.285 to 1.336)	p<0.001	1.339 (95% CI 1.311 to 1.368)	p<0.001
**Common Law Claims**	14,614 (12,478)	Crash Year	1.220 (95% CI 1.203 to 1.238)	p<0.001	1.229 (95% CI 1.211 to 1.248)	p<0.001
**All Claims**	200,831 (23,480)	Crash Year	1.124 (95% CI 1.120 to 1.129)	p<0.001	1.259^(95% CI 1.251 to 1.267)	p<0.001

* Adjusted for crash type, length of hospital stay, gender, age, SES, and remoteness

^ Further adjusted for claim type (no fault claims, no-fault impairment lump sum claims, and common law claims)

## Discussion

### Summary of findings

In 2013, one-fifth (20.1%) of TAC claims involved claimant lawyer use. Across the three groups, common law claims had the highest proportion of lawyer use, followed by no-fault impairment lump sum claims and, finally, no-fault claims. The proportion of claims that involved lawyer use approximately doubled between 2000 and 2013, although patterns of change differed across groups. Descriptive analyses demonstrated that there were no increases in rates of crashes or hospitalisations from crashes that accounted for this increase. Regression analyses demonstrated that the increase was also not due to variations in crash (type), injury (severity), or person (gender, age, SES, remoteness) factors over time.

### Comparison to existing australian research

The proportion of claims that involved lawyer use in this study is consistent with that reported in other research examining experience of personal injury in the Australian population. In such research, personal injury is defined as a harm to an individual for which compensation can be claimed; this harm may arise from a road traffic crash, workplace injury, product fault, or other injury circumstance [53]. The Legal Australia-Wide (LAW) Survey examined the incidence of personal injuries and actions taken in response to these injuries in a sample of 20,716 Australians [54]. Of those who experienced personal injuries, 72.2 percent took action by seeking advice, whilst 8.8 percent took action without seeking advice, and 19.0 percent took no action. Of those who took action, 20.8 percent obtained advice from a lawyer. If we consider lodging a TAC claim to represent taking action, the proportion of those who took action and obtained advice from a lawyer in the current study (20.1%) is similar to that in the LAW Survey (20.8%). This suggests that there is a similar propensity toward obtaining advice from a lawyer between Victorians who lodge TAC claims for road traffic injuries and Australians who experience all types of personal injuries.

The increase in lawyer use reported in this study is also consistent with evidence from other Australian schemes. For example, Ernst & Young [55] reported on trends in claims for minor severity injuries that involved legal representation in the New South Wales (NSW) road traffic injury compensation scheme at a time when this primarily provided fault-based benefits. Minor severity injuries were defined as injuries classified as being of minor or unknown severity by insurer staff using the Abbreviated Injury Scale [56]. As such, the NSW sample is similar to the no-fault claims group in the current study, with a caveat that it may be limited to not-at-fault claimants. It is unclear how legal representation was defined in the NSW report. Ernst & Young [55] found that the number of minor severity injury claims with legal representation per quarter decreased from 2001 to 2003, then remained stable until 2008, before increasing substantially between 2008 and 2015. Although the *proportion* of minor severity injury claims with legal representation was not reported, the number of claims with *no* legal representation declined between 2001 and 2009 before remaining stable, which is suggestive of a proportional increase in claims involving legal representation between 2008 and 2015. As such, the trend in NSW is similar to that in the current study, although the latter also found an increase in the number and proportion of claims involving lawyer use before 2008. The NSW report attributed the increase in minor injury severity claims involving legal representation to an increased propensity to claim in 2015 compared to 2008. However, in the current study, propensity to claim among those hospitalised for road traffic injuries appeared to decrease, not increase, during the study period.

### Increases in lawyer use overall

The increase in lawyer use in this study was not due to changes in rates of crashes or hospitalisations from crashes, or variations in observed crash, injury, or (measured) person factors over time: as such, it is likely to be due to other factors. These could include changes in: person factors not included in this study, such as legal consciousness (as greater legal consciousness is associated with greater capacity to resolve legal problems alone, without legal assistance; [54]); compensation scheme factors, such as the complexity and length of the claims process (as more complex and protracted processes are associated with greater legal service use; [14, 16, 57]); societal factors, such as attitudes towards lawyers and lawyer use (as social stigma and shame can deter claimants from engaging legal services; [58]); and regulation factors, such as legislation (as, for example, new requirements to resolve legal issues in a cost-effective, efficient, just, timely manner could increase the appeal of lawyer use; [59]). These could also include changes in the legal services market, such as increases in the number of lawyers operating in this market (as claimants are more likely to use legal services when these are readily available; [60]), and the proliferation of advertising by these lawyers (as this raises claimant awareness about the possibility of engaging legal services, and the process for doing so, as well as addressing barriers to, and reinforcing benefits of, use; [61, 62]).

In the absence of a concrete explanation for the increase in lawyer use, and a measure of its effect on claimant outcomes, implications for claimants and other schemes are difficult to determine. In terms of implications for claimants, given the instrumental role of lawyers in enabling claimants to access their legal entitlements, increased lawyer use could indicate greater claimant access to entitlements in the TAC scheme. However, if the increase in lawyer use was driven by changes in the scheme that made it more difficult for claimants to access entitlements without legal representation, this might not be the case. In terms of implications for other schemes, if the increase in lawyer use was driven by changes in local factors (such as insurer practices or the number of lawyers in Victoria) then the trend might not hold in other schemes. However, if the increase was driven by changes in global factors (such as the proliferation of advertising) then the trend might hold in other schemes.

### Increases in lawyer use in different claimant groups

In terms of the differences in patterns of change across groups, the minimal increase in the proportion of common law claims that involved lawyer use is likely to be due to a ceiling effect. This group had a high initial proportion of lawyer use, perhaps because of the complexity and perceived adversarial nature of the common law claiming process [14, 16]. Notably, although the common law claims group and the no-fault impairment lump sum claims group contained the greatest proportions of lawyer use, the actual numbers of claims in these groups were small. In contrast, the no-fault claims group contained the largest actual number of claims and, as a result, the increase in lawyer use seen in this group has the greatest implications for long-term scheme viability (given that the TAC is often liable for a proportion of claimants’ legal costs). The increase in lawyer use seen in this group also has implications for other jurisdictions that are considering, or are in the process of, reforming their compensation schemes to incorporate no-fault components. One common argument in favour of such reform is an anticipated reduction in legal costs, which are higher in fault-based systems due to the need to establish negligence [13, 32]. This study indicates that a non-trivial number and proportion of no-fault claims still involve lawyer use, and that this number and proportion are increasing. This suggests that reforming schemes to incorporate no-fault components might not affect legal costs as much as anticipated, particularly if the increase is being driven by global factors and continues into future years.

The increase in the proportion of no-fault claims that involved lawyer use may also have ethical and practical implications for lawyers. In these claims, lawyers are most often engaged to resolve disputes between claimants and the TAC (for example, over continued access to no-fault benefits) or lodge applications for impairment lump sum benefits that are later denied (for example, because the impairment threshold is not reached). If the increase in lawyer use in this group has been driven by increases in unsuccessful applications for impairment lump sum benefits, this may raise ethical questions, as lawyers are obligated to act only in cases with reasonable prospects of success [63]. If lawyers act in cases without such prospects, this can disadvantage claimants (through unnecessary exposure to harmful legal processes), insurers (through the cost of defending these cases), and courts (through wasted time and other resources) [64, 65]. It can also lead to unjust outcomes as, for example, insurers might choose to settle cases to avoid the costs of defending them [66]; this is important, given that lawyers’ ethical duties are first and foremost to the administration of justice [65]. If the increase in lawyer use in this group is due to increases in unsuccessful applications for impairment lump sum benefits, this also has practical implications, as the no-win-no-fee arrangements that are commonly used in personal injury claims in Australia mean that lawyers are not paid unless claims are successful [67]. The findings suggest that lawyers either may not be screening cases as effectively as they could be to select those with the best prospects of success, or may be assuming cases with greater risks of unsuccessful outcomes due to increased competition in the legal marketplace. In either case, lawyers may be screening out some claims that are legitimate, and pursuing others that are not, causing a disconnect between entitlement and award. However, it is unclear whether the increase in lawyer use in this group is due to an increase in unsuccessful impairment lump sum lodgements, or disputes.

### Limitations

This study has several limitations. First, there were differences in the measurement of hospitalisation across datasets, as TAC data were limited to admissions within one week of injury whilst VISU data were not. As a result, the lower number of TAC claims involving hospitalisations may be the result of discrepancies in measurement rather than true rates of hospital attendance. Second, the study may have overestimated the number of no-fault impairment lump sum claims, and under-estimated the number of common law claims, in the final years of the study period. This is due to the length of the common law claims process, which leads some claimants to lodge an impairment lump sum claim to receive an initial payout before a common law claim is lodged, or as a gateway to common law. As such, some no-fault impairment lump sum claims may later become common law claims. Third, lawyer use was underestimated in the regression analyses due to use of the limited sample with a four-year follow-up period (as this captured 85% of lawyer use). Fourth, this study focused on claimant lawyer use, as data about insurer lawyer use was unavailable; thus, not all lawyer use in the Victorian scheme has been captured. Fifth, the study is limited to compensable road traffic injury in Victoria; further research is needed to establish trends in lawyer use in other schemes.

Finally, restricting the sample used in the regression analyses to claims with lawyer use within four years post-crash introduced bias, as these were more likely to be made by claimants who were female, older, socio-economically advantaged, injured in multi-vehicle crashes, and severely injured than claims with lawyer use after four years post-crash. Notably, the LAW Survey found that individuals who take action (including legal action) in response to legal problems are more likely to be female, older, and have markers of socio-economic advantage than those who do not [54]. This study found that although legal services were used more frequently by claimants who were male, younger, and socio-economically disadvantaged, they were used *earlier* by claimants who were female, older, and socio-economically advantaged.

## Conclusions

This study provides an important initial look at lawyer use as an outcome in compensation systems. The results demonstrate that at present, one fifth of claims in the Victorian road traffic injury compensation scheme involve claimant lawyer use, a proportion that has steadily increased since 2000. Further research is needed to understand the predictors of lawyer use and reasons for this change, given the implications of increases in lawyer use for claimants (in terms of access to entitlements and justice), legal practitioners (in terms of ethics and practice), and compensation schemes (in terms of long-term scheme viability). Further research is also needed to understand the associations between lawyer use and claimants’ occupational, personal, and social functioning, in order to understand the implications of increases in lawyer use for claimant outcomes.

## Supporting information

S1 Appendix (DOCX)
