# Supplementary material for: Trends in lawyer use in road traffic injury compensation claims
Source: PLoS One. 2020 Apr 6;15(4):e0231025. doi: 10.1371/journal.pone.0231025 (PMC7135282; doi:10.1371/journal.pone.0231025)
Supplement: S1 Appendix — (DOCX) [file pone.0231025.s001.docx]

**Appendix A**

**Table A1. Multivariate Logistic Regression Results (Full Models)**

|  | **Group** | | | |
| --- | --- | --- | --- | --- |
| **Variable** | **No-Fault Claims** | **No-Fault Impairment Lump Sum Claims** | **Common Law Claims** | **All Claims** |
|  | **OR [95% CI]** | **OR [95% CI]** | **OR [95% CI]** | **OR [95% CI]** |
|  |  |  |  |  |
| **Crash Year** | 1.259 [1.249 - 1.269] | 1.339 [1.311 - 1.368] | 1.229 [1.211 - 1.248] | 1.259 [1.251 - 1.267] |
|  |  |  |  |  |
| **Crash Type (ref=Single Vehicle Crash)** |  |  |  |  |
| Multi Vehicle Crash | 1.014 [0.964 - 1.066] | 1.111 [0.968 - 1.275] | 1.613 [1.458 - 1.785] | 1.115 [1.069 - 1.163] |
|  |  |  |  |  |
| **Length of Hospital Stay (Days) (ref=0)** |  |  |  |  |
| 1 | 0.972 [0.906 - 1.044] | 0.607 [0.434 - 0.849] | 2.422 [1.976 - 2.968] | 1.110 [1.045 - 1.179] |
| 2 to 7 | 2.410 [2.257 - 2.574] | 0.413 [0.320 - 0.534] | 2.522 [2.193 - 2.900] | 2.419 [2.287 - 2.559] |
| More Than One Week | 7.124 [6.628 - 7.657] | 0.302 [0.242 - 0.376] | 3.791 [3.364 - 4.272] | 4.215 [3.967 - 4.478] |
|  |  |  |  |  |
| **Gender (ref=Male)** |  |  |  |  |
| Female | 0.931 [0.887 - 0.978] | 1.163 [1.011 - 1.337] | 1.183 [1.069 - 1.309] | 0.996 [0.955 - 1.037] |
|  |  |  |  |  |
| **Age Group (ref=35 to 44)** |  |  |  |  |
| Younger than 15 | 0.461 [0.406 - 0.522] | 0.447 [0.279 - 0.717] | 0.454 [0.346 - 0.594] | 0.459 [0.410 - 0.514] |
| 15 to 24 | 0.491 [0.454 - 0.532] | 1.009 [0.810 - 1.257] | 0.892 [0.758 - 1.049] | 0.569 [0.532 - 0.608] |
| 25 to 34 | 0.740 [0.686 - 0.798] | 1.100 [0.889 - 1.360] | 1.024 [0.879 - 1.193] | 0.807 [0.757 - 0.861] |
| 45 to 54 | 1.117 [1.034 - 1.206] | 1.022 [0.818 - 1.278] | 1.082 [0.931 - 1.258] | 1.124 [1.051 - 1.201] |
| 55 to 64 | 0.950 [0.870 - 1.038] | 0.795 [0.622 - 1.016] | 1.259 [1.054 - 1.504] | 1.010 [0.937 - 1.089] |
| 65 to 74 | 0.711 [0.637 - 0.794] | 0.588 [0.448 - 0.771] | 1.736 [1.347 - 2.238] | 0.827 [0.755 - 0.905] |
| 75 and older | 0.440 [0.391 - 0.495] | 0.319 [0.236 - 0.431] | 1.548 [1.120 - 2.139] | 0.533 [0.482 - 0.589] |
|  |  |  |  |  |
| **SES: IRSAD State Decile (ref=Deciles 5 and 6)** |  |  |  |  |
| Deciles 1 and 2 | 1.216 [1.129 - 1.309] | 1.463 [1.187 - 1.803] | 1.113 [0.956 - 1.297] | 1.231 [1.156 - 1.311] |
| Deciles 3 and 4 | 1.071 [0.990 - 1.158] | 1.123 [0.911 - 1.383] | 0.900 [0.767 - 1.056] | 1.042 [0.975 - 1.114] |
| Deciles 7 and 8 | 0.828 [0.766 - 0.895] | 0.932 [0.758 - 1.147] | 0.903 [0.770 - 1.059] | 0.855 [0.801 - 0.913] |
| Deciles 9 and 10 | 0.822 [0.763 - 0.885] | 0.827 [0.670 - 1.021] | 0.877 [0.746 - 1.030] | 0.824 [0.774 - 0.878] |
|  |  |  |  |  |
| **Remoteness (ref=Major Cities of Australia)** |  |  |  |  |
| Inner Regional Australia | 0.775 [0.727 - 0.826] | 0.800 [0.680 - 0.942] | 0.770 [0.680 - 0.871] | 0.757 [0.718 - 0.799] |
| Outer Regional, Remote, and Very Remote Australia | 0.987 [0.885 - 1.101] | 0.537 [0.405 - 0.713] | 0.825 [0.664 - 1.024] | 0.867 [0.787 - 0.954] |
|  |  |  |  |  |
| **Claim Type (ref=No-Fault Claims)** |  |  |  |  |
| No-Fault Impairment Lump Sum Claims | - | - | - | 26.400 [24.524 - 28.418] |
| Common Law Claims | - | - | - | 130.689 [123.038 - 138.816] |
